# Patient Safety Culture from a Nursing Perspective in a Chilean Hospital

**DOI:** 10.3390/nursrep14020108

**Published:** 2024-06-04

**Authors:** Paulina Hurtado-Arenas, Miguel R. Guevara, Víctor M. González-Chordá

**Affiliations:** 1 School of Nursing, Universidad de Valparaíso, 2540064 Valparaíso, Chile; 2 Data Science Laboratory, Department of Computer Science, Faculty of Engineering, Universidad de Playa Ancha, 2360002 Valparaíso, Chile; miguel.guevara@upla.cl; 3 Nursing Research Group (GIENF Code 241), Nursing Department, Universitat Jaume I, 12071 Castellón de la Plana, Spain; vchorda@uji.es; 4 Nursing and Healthcare Research Unit (INVESTÉN-ISCIII), Institute of Health Carlos III, 28029 Madrid, Spain; 5 Network Biomedical Research Center on Frailty and Healthy Aging (CIBERFES), Institute of Health Carlos III, 28029 Madrid, Spain

**Keywords:** patient safety, organizational culture, nursing staff, hospitals

## Abstract

Patient safety culture is relevant both in the delivery of care and in the training of nursing staff, its purpose being to prevent and reduce risks associated with health care. This research aims to evaluate patient safety culture from the perspective of the nursing teams in a highly complex public hospital in the city of Valparaíso, Chile. A cross-sectional study with a quantitative approach applying descriptive, bivariate, and inferential statistical analysis was conducted on 259 nurses and nursing assistants from 13 adult medical–surgical units of the Carlos Van Buren hospital. The participants were obtained through a non-probabilistic convenience sample, answering the hospital survey on Patient Safety Culture version 2.0 (HSOPS 2.0), adapted to the Chilean population. The best-evaluated dimension was communication and receptivity; the worst was the support administrators provide for patient safety. This study identified the weaknesses and strengths of the hospital, the most worrying weakness being the shortage of human capital, material, and financial resources necessary to improve patient safety. This study was not registered.

## 1. Introduction

The World Health Organization (WHO) states that patient safety is a global health priority, emerging with the evolution of the complexity of healthcare systems and the increase in harm to patients in healthcare facilities. Considering the systemic approach to patient safety, most patient harm is not due to incorrect practices on the part of healthcare personnel but is rather the product of systemic or procedural inconsistencies that trigger such human errors [1]. These errors are a consequence of existing deficiencies in the structures and processes of the system with complex environments, including the stress placed on health personnel, especially nurses, when providing care [2]. The role played by the behavior of nursing staff in resolving incidents, together with the organizational culture and work environment, are aspects to consider when implementing measures to improve patient safety [3]. Recognizing the crucial role of nurses, researchers have proposed a nursing-specific definition of patient safety that includes three key components: the meaning of harm for the patient, interventions to eliminate or reduce this harm, and identification of areas of nursing practice that pose risks to the patient. Patient safety in nursing is defined as the state in which harm caused to the patient by nursing practice is eliminated or reduced to the greatest extent possible through a continuous process of identifying adverse events [4].

In this context, and following the recommendations of the global action plan for patient safety 2021–2030 [5], patient safety culture (PSC) acquires excellent relevance both in care delivery and in nursing staff training to prevent and reduce the risks associated with health care [6]. Patient safety culture encompasses a set of values, attitudes, perceptions, and individual or group competencies that influence the management of safety in the care provided to patients. Its aim is to ensure that patients do not experience unnecessary harm or potential risks associated with their healthcare [7].

Evaluating patient safety culture provides information to nurse managers to develop strategies to improve the quality of care. The Agency for Healthcare Research and Quality (AHRQ) [8] developed instruments to evaluate patient safety culture, highlighting the Hospital Survey on Patient Safety Culture (HSOPS), widely used in version 1.0 and version 2.0 [9]. Version 1.0 was created in 2004 and comprises 42 items organized into 12 dimensions. Meanwhile, version 2.0 was created in 2019 and has ten dimensions and 32 items, the latter being more focused and direct in delivering more reliable results. HSOPS 2.0 has been validated in countries such as China [10], Korea [11], Turkey [12], Indonesia [13], and Brazil [14]. A version of the HSOPS 2.0 has been adapted for the Chilean context, although the results still need to be published.

At an international level, a study related to China mentions that the culture of patient safety in health institutions is validated as one of the pillars of preventive strategies that ensure quality of care with the participation of nursing staff in patient safety training [15]. In South Korea, it is emphasized that prioritizing patient safety, encouraging transparent communication, and promoting a reporting culture cultivates a learning environment within the organizational culture, ultimately enhancing care quality [16]. Another study related to Turkey highlights the responsibility of managers in the culture of patient safety since providing an environment where errors are discussed without fear of punishment will improve the work environment and, therefore, the quality of the care supplied [17]. A systematic literature search study aimed at understanding the evolution of patient safety culture in Western countries, such as Australia, England, Canada, and the United States, highlights the fundamental contribution of specialized nurses’ competencies in establishing and reinforcing a culture of patient safety in nursing, both in hospitals and in outpatient care [18]. Within the scope of Latin America, a study related to Brazil states that patient safety is directly related to the quality of the processes and prevention of infections associated with health care in nursing teams, negatively highlighting the factors of difficulty in internal communication and learning from mistakes [19]. Currently, in Chile, the National Health Strategy of the Ministry of Health (MINSAL) points out four health objectives for 2021–2030, among which the highlight is to ensure the quality of health care, where one of its strategic axes is quality of health [20].

Considering the importance of patient safety culture and the influence of nursing staff on the quality of care provided, the development of research that measures patient safety culture in the hospital setting is relevant. With a background indicating that Chile does not have studies that reveal patient safety culture in the nursing staff of public hospitals by using the HSOPS 2.0 adapted to the Chilean population and that it is relevant to evaluate it to contribute to the continuous improvement of quality, this research aims to assess the culture of patient safety from the perspective of the nursing teams in a highly complex public hospital in the city of Valparaíso, Chile.

## 2. Materials and Methods

### 2.1. Type of Study

A cross-sectional study with a quantitative approach that employs descriptive, bivariate, and inferential statistical analyses was carried out on 13 adult medical–surgical units at the Carlos Van Buren public hospital in Valparaíso, Chile, from May to July 2021. Only medical–surgical units authorized by the public hospital were considered for this study.

### 2.2. Population and Sample

The study population comprised all nursing staff (nurses and nursing assistants) at the Carlos Van Buren Hospital (N = 370). Nursing assistants were included because they play a vital role within each nursing team, along with nurses, in providing care to patients in Chile. In medical–surgical units, they have the responsibility of providing basic nursing care to the hospitalized person, such as hygiene and comfort, control of vital signs, administration of medication, administration of oxygen, and prevention of pressure ulcers, among others, always acting under the supervision of the charge nurse. 

More than three months of work experience at the Carlos Van Buren Hospital was considered an inclusion criterion, and exclusion criteria were considered for the administrative staff who did not provide direct patient care. Participants were obtained through non-probability convenience sampling, with an established minimum response rate of 60%, which is acceptable [21], and was conditioned for the period when the data were gathered (the COVID-19 pandemic).

### 2.3. Variables and Instruments

Sociodemographic variables such as age and sex were collected. In addition, work variables such as type of staff, hospitalization unit, level of training, length of contract, weekly working hours, and contact with the patient were also collected.

Safety culture was measured with a version of the HSOPS 2.0 questionnaire adapted to the Chilean population, the results of which are pending publication. Translation and cross-cultural adaptation, content validity through a group of experts, and a pilot test with a cognitive pretest were applied. Compared with the original instrument, the adapted questionnaire did not include new items but reduced the number of dimensions from 10 to 7 and the number of items from 32 to 23. This new structure was determined after an exploratory and confirmatory analysis. Additionally, all questions were worded positively. Its psychometric properties, such as content validity (S-CVI global content validity index > 0.982) [18], construct validity [22] (RMSEA = 0.048, SRMR = 0.064; $\chi^{2}/df=1.46$), and reliability measured with McDonald’s Omega coefficient (ω=0.9325) were considered adequate. The HSOPS 2.0 adapted to the Chilean population can be consulted in Supplementary Material Table S1.

This Chilean version of the HSOPS 2.0 questionnaire comprises 23 items organized into seven dimensions: D1—Teamwork and response to errors, D2—Staffing and organizational learning, D3—Supervisor support for patient safety, D4—Communication regarding errors, D5—Communication and responsiveness, D6—Reporting events related to patient safety patient, and D7—Support that administrators give for patient safety. In addition, it requests a general patient safety evaluation and allows for voluntary comments at the end of the questionnaire. The Likert scale used for each statement graduated from 1 to 5. 

### 2.4. Data Collection

The data were collected between May and July 2021, responding to the Hospital Survey on Patient Safety Culture version 2.0 (HSOPS 2.0), adapted to the Chilean population. To achieve this, an email from the hospital’s nursing management sub-direction sent a Google form. The online form initially incorporated an informed consent form that indicated voluntary and anonymous participation. The nurses and nursing assistants were trained through group conferences via the Zoom platform, and individual explanatory digital capsules were distributed via email and WhatsApp.

The response time was 60 days, and a weekly reminder was sent. Forms that were not answered in their entirety were excluded.

### 2.5. Analysis

First, a descriptive analysis of the sociodemographic and work variables of the sample was carried out. Quantitative variables were described by mean and standard deviation. The qualitative variables were analyzed using frequency distribution and percentages.

The results on safety culture were analyzed following the HOSPS 2.0 User Guide. According to this guide, values 4 and 5 are classified as positive, while values 1 and 2 are classified as negative. Values 3 are classified as neutral. The total number of responses and the relative frequency of positive, negative, and neutral responses, with their corresponding percentages, were calculated for each question. Empty values were excluded. The average rate for positive, negative, and neutral responses was calculated for each dimension based on their corresponding items.

Furthermore, the questionnaire results were compared based on socio-demographic and work variables with the non-parametric Mann–Whitney test in the case of two groups or with the Kruskal–Wallis test in the case of three or more groups. It was previously confirmed that the sample did not meet the conditions for applying the parametric tests. A significance level of *p* < 0.05 was considered in the hypothesis tests.

A linear regression model was used for inferential analysis. The dependent variable was defined as the general patient safety evaluation reported by each participant. Seven independent variables were considered by summing the values of responses in each dimension. Incomplete data were excluded from the analysis.

Data analysis was performed with R statistical software, version 4.3.0. 

### 2.6. Ethical Considerations

This study was favorably evaluated by the Scientific Ethics Committee of the Valparaíso San Antonio, Chile Service on 21 August 2019, with file 04/2019, and by the Ethics Commission of the Universitat Jaume I on 13 September 2019, with file CD/43/2019.

In addition, the ethical considerations provided for in Law 20,585 on access to the public information of Chile [23] were considered, as were the principles of the Declaration of Helsinki [24], which include social value, scientific validity, equitable selection of the participant, a favorable risk–benefit relationship, independent evaluation, informed consent, and respect for enrolled participants.

## 3. Results

### 3.1. Sample Characterization

A total of 259 responses were obtained, and none were excluded, which is equivalent to a response rate of 70%, overcoming the 60% set as the minimum required. The sample comprised 56.4% (n = 146) nurses and 43.6% nursing assistants (n = 113). The age range was 19 to 63 years, with the median at 33 years and a standard deviation of 9.86. People from 13 units of the hospital participated. Regarding sex, 90% of the participants were women (n = 233), 9.3% were men, and 0.8% declared another option. Only 5% (n = 13) of nurses had a master’s degree, 20.5% of nurses (n = 53) had a diploma, and 3.9% of nurses (n = 10) had a specialty. Regarding seniority in the unit, 25.1% (n = 65) had worked for less than one year, 44.4% (n = 115) had worked between 1 and 5 years, 14.7% (n = 38) had worked from 6 to 10 years, and 15.8% had worked in the hospital for 11 or more years. In total, 92.3% of the participants worked in contact with patients. Table 1 presents details regarding the characterization of the sample.

### 3.2. Descriptive Results for Each Dimension of Safety Culture

The following results for each item and overall dimension are presented in Table 2. 

Regarding the results obtained for Dimension 1—Teamwork and response to errors, it was noted that item A1, “In this unit, we work as a team efficiently”, had the highest percentage of positive responses (84.9%). On the other hand, item A10, “When staff make mistakes, this unit concentrates more on learning from them than on finding blame”, had the most negative options (14.6%). 

For Dimension 2—Staffing and Organizational Learning, the item with the highest percentage of positive responses was item A3, “In this unit, the staff can perform adequately in patient care”. With 78.4%, item A2, “In this unit, we have enough personnel to do all the work” obtained 41.3% negative responses, the highest negative value of the 23 questions in the instrument.

Regarding the results obtained for Dimension 3—Support given by supervisors for patient safety, item B1, “My supervisor considers staff suggestions that seek to improve patient safety”, received the best positive responses, 80.2%. By contrast, item A12, “In this unit, the changes made to improve patient safety are periodically evaluated to see how well they are working”, obtained results with the highest negative responses (12.1%).

For Dimension 4—Communication regarding errors, the highest proportion of positive responses occurred in item C3, “In this unit, we are communicated about improvement decisions that are made based on reported adverse events” with 79.1%. In comparison, 6.8% of negative responses occurred in item C2, “When there is an adverse event in this unit, we analyze ways to prevent it from happening again”. 

Concerning Dimension 5—Communication and receptivity, the highest positive values occurred in item C4, “In this unit, the staff reports if they see something that could harm the patient’s care”. With 92.6% of positive responses, this was also the highest value of the 23 items that the instrument included.

Regarding the results for Dimension 6—Report events related to patient safety, item D2, “When an error affects the patient without causing harm, how often is it reported?” obtained 86.4% positive responses, and the other item of the dimension, D1, “When an error is identified and corrected before the patient is affected, how often is it reported”? obtained the highest negative responses with 4.4%.

Finally, regarding the results of Dimension 7—Support that administrators give for patient safety, item F1, “The mission of the hospital demonstrates that patient safety is paramount” obtained 70.2% positive responses. By contrast, item F2, “Hospital management provides human capital, material, and financial resources necessary to improve patient safety” obtained the highest percentage of negative responses in this category with 3.73%.

### 3.3. Overall Results

The results, in general, identified three dimensions with percentages of average positive responses greater than or equal to 75%. These were dimensions D3—Support provided by supervisors for patient safety (76%), D5—Communication and receptivity (84.4%), and D6—Report events related to patient safety (83.8%).

In turn, Dimension 7, Support given by administrators for patient safety, was detected as a dimension with low positive responses, with only 50.7% positive and 20.1% negative responses. However, the most significant number of neutral responses was also detected in this dimension, with an average of 29.2% of the total responses to the items that comprised this dimension. Table 3 presents the summary of average values obtained for each dimension.

Table 4 presents the results of responses regarding questions that account for the number of events reported and the general perception of patient safety culture.

For the bivariate analysis, the questions were added, considering only complete answers. Table 5 presents the results of this analysis, showing significant differences between the groups in their role within the nursing teams, their work experience duration in the unit, their working hours per week, and the number of reported events.

### 3.4. Inferential Analysis

Table 6 presents the results for a linear regression model, considering the self-reported general patient safety evaluation (see Table 4) as the dependent variable and the score obtained in each dimension (adding each dimension’s item) as seven independent variables. The results obtained are as follows: Residual standard error = 0.5948 on 187 degrees of freedom; Multiple R-squared = 0.4544; Adjusted R-squared = 0.434; F-statistic = 22.25 on 7 and 187 DF; and *p*-value: <2.2 × 10^−16^.

It can be observed that D2—Staffing and Organizational Learning (*p* = 1.1 × 10^−5^) is highly significant, suggesting a strong relationship with the dependent variable. D3 (*p* = 0.0158) is also significant, and D5 (*p* = 0.0599) is near significant. These values indicate that approximately 45.44% of the variance in self-reported patient safety is explained by the model. The F-statistic tests measuring whether at least one of the predictor variables is significantly related to the dependent variable were significant. The very low *p*-value (<2.2 × 10^−16^) indicates that the model is statistically significant overall.

## 4. Discussion

No other study in Latin America uses version 2.0 of the HSOPS adapted to its country’s population. This converges with what was expressed in a study published in 2023 by Pedroso and collaborators [25], which highlights the importance of advancing the implementation of permanent measurements of this area in public health. Most of the discussions in Latin America are carried out by studies that applied version 1.0 of the HSOPS instrument, which was later refined to a reduced number of items and categories in version 2.0.

The descriptive results of this study consider the focus on a highly complex hospital, with the participation of a majority of female nurses with work experience of fewer than five years, similar to research developed in Colombia [26] and Brazil [27]. Most of the participants in this research were in an age range of 19 to 63 years old and worked more than 40 h a week, which aligns with another study in Minas Gerais, Brazil [28]. Population factors, such as lack of knowledge of patient safety culture, work overload, fear of retaliation, and punishment within the work environment, can explain the long workdays and underreporting of events noted in this study [29]. The description of postgraduate and specialty nursing studies is not seen in other studies since none mention this category when describing their sample. 

The dimensions Communication and receptivity and Report events related to patient safety obtained higher positive responses. Thus, the staff valued communication positively. However, questions linked to an authority presented lower values, which reaffirms the “fear” of authority seen in countries in Latin America, which is often caused by job insecurity and the fear of losing one’s job [25].

Teamwork and response to errors did not present highly positive responses. However, teamwork was valued positively, as seen, for example, in question A1, “In this unit, we work as a team efficiently” and in question A9, “The members who work in this unit treat each other with respect”. In this same dimension, reporting and learning carried out as a team was evaluated with low values, which can be seen in question A7, “In this unit, when an incident is reported, the report focuses on the problem and not about the staff” and A10, “When staff make mistakes, this unit focuses more on learning from them than on finding blame”. With the above, we see the need to reinforce how teams deal with possible errors. These results coincide with a multicenter study in South America [25], emphasizing teamwork as an item valued positively with the highest scores.

The dimension with the worst rating concerns administrators. This shortcoming is usually linked to the scarce resources allocated to public health in Chile [30]. In an environment where resources are scarce, security is not a priority.

The above dimension of Staffing and Organizational Learning, which asks if there is enough staff to carry out all the work, must also be observed critically since data collection was carried out during the COVID-19 pandemic when staffing was scarce [31].

Regarding the general perception of patient safety in the work unit, the participants of this study rated it as very good and excellent, coinciding with another study from Mexico [32] that reported 50% as very good and agreed that there were opportunities for improvement.

Regarding the perception of the patient’s safety culture analyzed based on socio-demographic and work variables, statistically significant differences were observed in terms of the role within the nursing teams, work experience duration in the hospital, and in the unit concerning weekly time commitments and events reported in the last twelve months. Regarding the role within the nursing teams, nurses gave a higher assessment compared to nursing assistants; this may be because nurses have greater access to strengthening their professional leadership competence, teamwork, and assertive communication, as well as acquiring more relevant knowledge to provide quality care in patient safety, using tools specific to the discipline such as the nursing process [33].

Regarding work experience duration in the hospital and the unit, an interesting finding emerges; participants with less than one year and those with more than 11 years of work experience tended to value the culture of patient safety. This fact could be associated with teamwork between novices and experts to positively value patient safety, coinciding with a study conducted in a hospital in Valencia, Spain [34]. Regarding time commitment, the group with the most minor weekly rostered hours (less than 30 h a week) valued patient safety culture more positively. In the number of events reported in the last 12 months, it is evident that those who do not report incidents and those who report more incidents (11 or more) rate them more positively, with a median equal to 88.

The inferential analysis reported a statistically significant model overall. However, it explains only 45.44% of the variance in the general perception of patient safety. Further research should be oriented toward proposing models that associate results in each dimension of the questionnaire with measured indicators of patient safety, such as the number of adverse events [35]. 

The results of this study should be considered with caution. On the one hand, it is a study carried out in a single center with a non-randomized sample, which makes it difficult to generalize the results. In addition, participation was affected by the SARS-CoV-2 pandemic and the workload at that time; a response rate higher than expected was obtained despite this. Even so, the results of this study are considered relevant due to the lack of studies that measure patient safety culture with validated instruments in Latin America and, specifically, Chile. The results of this study are also helpful in guiding the decision making of nurse managers and decision makers regarding strategies to improve safety culture in this context.

## 5. Conclusions

The evaluation of patient safety culture from the perspective of the nursing teams in a highly complex public hospital in Latin America made it possible to identify weaknesses and strengths in the health institution. The most worrying weakness is providing human capital, resources, materials, and finances necessary to improve patient safety by hospital management.

Applying the adapted version of HSOPS 2.0 to the Chilean population is the first step in promoting effective institutional changes. Hospital management should allocate human capital, resources, and funding to implement specific strategies that support establishing a culture of patient safety. 

This culture should be based on a safe and non-punitive environment for reporting adverse events. Additionally, it is essential to provide ongoing education for nursing staff on patient safety and to develop behavioral guidelines on the subject, among other measures.

Based on this study’s findings, it is suggested that a more significant number of closed-care public or private health establishments in the country replicate this study for future research to meet the health objective for 2021–2030, which is related to ensuring healthcare quality.

## Figures and Tables

**Table 1:** Descriptive characterization of the sample.

Characteristic	Option	Frequency	Percentage
Type of staff	Nursing assistant	113	43.6
	Nurse	146	56.4
Hospitalization unit	1. Many different hospital units, without a specific unit	3	1.2
	2. Low Medical Complexity—Medicine (seventh floor)	41	15.8
	3. Low Medical Complexity—Oncology	26	10
	4. Low Surgical Complexity—Neurosurgery	16	6.2
	5. Low Surgical Complexity—Pensioner	3	1.2
	6. Acute Medical Unit (UMA)	11	4.2
	7. Low Complexity Medical Surgical—Ophthalmology	12	4.6
	8. Low Complexity Medical Surgical—Otorhinolaryngology	11	4.2
	9. Medium Surgical Complexity	16	6.2
	10. Medium Medical Complexity	14	5.4
	11. General Intensive Care Unit	18	6.9
	12. General Medical Intermediate Care Unit	36	13.9
	13. COVID-19 Intensive and Intermediate Medial Care Unit	52	20.1
Sex	Man	24	9.3
	Woman	233	90
	Other	2	0.8
Nurse Postgraduate	Master	13	5
	None	246	95
Nurse Specialization	Diploma	53	20.5
	Specialty	10	3.9
	None	196	75.7
Hospital seniority	Less than 1 year	44	17
	From 1 to 5 years	101	39
	From 6 to 10 years	49	18.9
	11 or more years	65	25.1
Unit seniority	Less than 1 year	65	25.1
	From 1 to 5 years	115	44.4
	From 6 to 10 years	38	14.7
	11 or more years	41	15.8
Weekly working hours (hours)	30 to 40 h a week	30	11.6
	More than 40 h a week	223	86.1
	Less than 30 h a week	6	23
Direct contact with patients	NO, I normally DO NOT have direct interaction or contact with patients.	20	7.7
	YES, I normally have direct interaction or contact with patients.	239	92.3

**Table 2:** Results of each item and dimension.

Item	Question	Total	Positive (%)	Neutral (%)	Negative (%)
A1	In this unit, we work as a team efficiently.	259	**0.849**	0.112	0.039
A8	In this unit, we work collaboratively, independent of the existing workload.	258	0.744	0.174	0.081
A9	The members who work in this unit treat each other with respect.	256	0.824	0.105	0.070
A7	In this unit, when an incident is reported, the report focuses on the problem and not the personnel.	247	0.656	0.194	0.150
A10	When staff make mistakes, this unit focuses more on learning from them than on finding blame.	253	0.648	**0.206**	**0.146**
D1 Averages: Teamwork and response to errors		254.60	0.744	0.158	0.097
A2	In this unit, we have enough staff to do all the work.	259	0.347	**0.239**	**0.413**
A3	In this unit, the staff can perform adequately in patient care.	259	**0.784**	0.143	0.073
A4	This unit regularly reviews work processes and protocols to determine if changes are necessary to improve patient safety.	255	0.753	0.133	0.114
D2 Averages: Staffing and Organizational Learning		257.67	0.628	0.172	0.200
B1	My supervisor considers staff suggestions that seek to improve patient safety.	256	**0.809**	0.109	0.082
B3	My supervisor takes action to resolve patient safety issues that have been reported to him or her.	253	0.802	0.107	0.091
C6	When this unit staff makes a report, authorities are open to hearing your observations and concerns regarding patient safety.	244	0.742	0.164	0.094
A12	In this unit, changes made to improve patient safety are periodically evaluated to see how well they are working.	248	0.685	**0.194**	**0.121**
D3 Averages: Support given by supervisors for patient safety		250.25	0.760	0.143	0.097
C1	We are informed about adverse events that occur in this unit.	254	0.673	**0.268**	0.059
C2	When there is an adverse event on this unit, we look at ways to prevent it from happening again.	251	0.749	0.183	**0.068**
C3	In this unit, we are informed about improvement decisions that are made based on the reported adverse events.	254	**0.791**	0.142	0.067
D4 Averages: Communication regarding errors		253.00	0.738	0.198	0.065
C4	In this unit, staff report if they see something that could harm the patient’s care.	258	**0.926**	0.047	0.027
C5	When the staff of this unit sees that someone of higher authority is doing something that compromises the patient’s safety, they report it.	241	0.793	**0.174**	0.033
C7	The staff of this unit asks questions without fear when they observe a situation that compromises the patient’s safety.	258	0.814	0.116	**0.070**
D5 Averages: Communication and receptivity		252.33	0.844	0.112	0.043
D1	Think about your unit/work area. When an error is identified and corrected before the patient is affected, how often is it reported?	251	0.813	0.143	**0.044**
D2	Think about your unit/work area. When an error affects the patient without causing harm, how often is it reported?	250	**0.864**	0.108	0.028
D6 Averages: Report events related to patient safety		250.50	0.838	0.126	0.036
F1	The hospital’s mission demonstrates that patient safety is paramount.	252	**0.702**	0.242	0.056
F2	Hospital management provides human capital, material, and financial resources necessary to improve patient safety.	255	0.345	**0.282**	**0.373**
F3	Hospital management is interested in patient safety after an adverse event occurs.	**234**	0.474	0.350	0.175
D7 Averages: Support that administrators give for patient safety		247.00	0.507	0.292	0.201

* Values in **bold** represent the maximum value in that column for a particular dimension.

**Table 3:** General results by dimension.

	D1	D2	D3	D4	D5	D6	D7
Total responses	254.6	257.7	250.3	253.0	252.3	250.5	247.0
Positives	189.8	161.7	190.3	186.7	213.3	210.0	125.3
Positives %	0.744	0.628	0.760	0.738	0.844	0.838	0.507
Neutral	40.2	44.3	35.8	50.0	28.0	31.5	71.7
Neutral %	0.158	0.172	0.143	0.198	0.112	0.126	0.292
Negatives	24.6	51.7	24.3	16.3	11.0	9.0	50.0
Negatives %	0.097	0.200	0.097	0.065	0.043	0.036	0.201

**Table 4:** Results regarding reported events and general perception of patient safety.

In the Past 12 Months, How Many Patient Safety Events Have You Reported?			How Would You Rate Patient Safety in Your Unit/Work Area?		
	Frequency	Percentage		Frequency	Percentage
None	84	0.324	Deficient	0	0.000
1 to 2	96	0.371	Regular	8	0.031
3 to 5	47	0.181	Good	81	0.313
6 to 10	17	0.066	Very good	120	0.463
11 or more	15	0.058	Excellent	50	0.193

**Table 5:** Bivariate analysis.

Question	Category	N	Min.	1st Q	Medium	Mean	3rd Q	Max.	*p*_Value
What is your job at this hospital?	Nurse	118	49	86	95	93.25	102	115	0.02102 *
	Nurse Assistant	77	47	80.25	89.5	88.83	97.75	112	
Sex	Man	19	49	84.5	91	89.53	96.5	115	0.7059
	Woman	174	47	82.25	92	90.6	100.75	115	
	Other	2	91	94.75	98.5	98.5	102.25	106	
How long have you been working at this hospital?	Less than 1 year	30	68	89.25	97.5	96.43	107.5	115	0.02336 *
	From 1 to 5 years	83	54	81	88	88.48	96	115	
	From 6 to 10 years	36	49	79.75	87.5	88.92	99.25	110	
	11 or more years	46	47	86.25	93	91.83	101.75	112	
At this hospital, how long have you been working in your current unit/work area?	Less than 1 year	45	59	87	99	97.38	110	115	0.0007613 *
	From 1 to 5 years	96	54	80.75	88.5	88.11	96	115	
	From 6 to 10 years	24	49	79.25	89.5	87.83	99.75	108	
	11 or more years	30	47	86	92	90.43	100.75	110	
Typically, how many hours a week do you work at this hospital?	Less than 30 h a week	4	101	109.2	112.5	110.2	113.5	115	0.004416 *
	30 to 40 h a week	17	79	88	95	95.41	100	115	
	More than 40 h a week	174	47	81.25	91	89.65	98.75	115	
In your workplace, do you normally have direct interaction or contact with patients?	YES, I normally have direct interaction or contact with patients.	179	47	81.5	91	90.03	100	115	0.0656
	NO, I normally DO NOT have direct interaction or contact with patients	16	85	87.75	96.5	96.69	101	115	
In the past 12 months, how many patient safety events have you reported?	None	60	49	86	95.5	94.35	106	115	0.0008366 *
	3 to 5	31	50	83.5	87	88.26	101.5	115	
	6 to 10	12	47	71.75	79	76.75	83.25	101	
	11 or more	13	80	88	96	94,692	96	115	

* Significance level *p* < 0.05.

**Table 6:** Results of the linear regression model.

	Estimate	Std. Error	t Value	Pr(>|t|)	
(Intercept)	0.43125	0.31353	1.375	0.1706	
D1	0.14458	0.38897	0.372	0.7105	
D2	1.6417	0.36336	4.518	1.10 × 10^−5^	***
D3	0.95391	0.39157	2.436	0.0158	*
D4	0.42851	0.37044	1.157	0.2489	
D5	0.94769	0.50054	1.893	0.0599	.
D6	0.04177	0.38997	0.107	0.9148	
D7	0.25292	0.3338	0.758	0.4496	

Significance codes: 0 “***” 0.01 “*” 0.05 “.” 0.1 “ “ 1.

## Data Availability

Data are available upon reasonable request. All necessary data are supplied and available in the manuscript; however, the corresponding author will provide the dataset upon request. All data relevant to the study are included in the article.

## Supplementary Materials

The following supporting information can be downloaded at https://www.mdpi.com/article/10.3390/nursrep14020108/s1, Table S1: Proposal for a validated HSOPS 2.0 Instrument adapted to the Chilean population.

## Public Involvement Statement

There was no public involvement in any aspect of this research.

## Guidelines and Standards Statement

This manuscript was drafted against the STROBE for quantitative cross-sectional research.
