# Supplementary material for: Patient Safety Culture from a Nursing Perspective in a Chilean Hospital
Source: Nurs Rep. 2024 Jun 4;14(2):1439–51. doi: 10.3390/nursrep14020108 (PMC11206771; doi:10.3390/nursrep14020108)
Supplement: Supplementary file 1 [file nursrep-14-00108-s001.zip › nursrep-3010923-supplementary.pdf]

Supplementary material for Patient Safety Culture from a Nursing Perspective in a Chilean Hospital

Supplementary Table S1. Proposal for a validated HSOPS 2.0 Instrument adapted to the Chilean population

Item [1]	Description [2]
**Dimension 1: Teamwork and response to errors (Ex D1 Teamwork and Ex D4 Response to errors)**	
A1	In this unit, we work as a team efficiently. [Ex D1]
A8	In this unit, we work collaboratively, independent of the existing workload. [Ex D1]
A9	The members who work in this unit treat each other with respect. [Ex D1]
A7	In this unit, when an incident is reported, the report focuses on the problem and not the personnel. [Ex D4]
A10	When staff make mistakes, this unit focuses more on learning from them than on finding blame. [Ex D4]
**Dimension 2: Staffing and Organizational Learning (Ex D2 Pressure and work pace and Ex D3 Organizational learning)**	
A2	In this unit, we have enough staff to do all the work. [Ex D2]
A3	In this unit, the staff has the ability to perform adequately in patient care. [Ex D2]
A4	This unit regularly reviews work processes and protocols to determine if changes are necessary to improve patient safety. [Ex D3]
**Dimension 3: Support provided by supervisors for patient safety (Ex D5 Support provided by supervisors, directors or clinical heads for patient safety, Ex D7 Communication and Receptivity, Ex D3 Organizational learning and)**	
B1	My supervisor considers staff suggestions that seek to improve patient safety. [Ex D5]
B3	My supervisor takes action to resolve patient safety issues that have been reported to him or her. [Ex D5]
C6	When this unit staff makes a report, authorities are open to hearing your observations and concerns regarding patient safety. [Ex D7]
A12	In this unit, changes made to improve patient safety are periodically evaluated to see how well they are working. [Ex D3]
**Dimension 4: Communication regarding errors**	
C1	We are informed about adverse events that occur in this unit.
C2	When there is an adverse event on this unit, we look at ways to prevent it from happening again.
C3	In this unit, we are informed about improvement decisions that are made based on the reported adverse events.
**Dimension 5: Communication and receptivity**	
C4	In this unit, staff report if they see something that could harm the patient's care.
C5	When the staff of this unit sees that someone of higher authority is doing something that compromises the patient's safety, they report it.

Item [1]	Description [2]
C7	The staff of this unit asks questions without fear when they observe a situation that compromises the patient's safety.
**Dimension 6: Report events related to patient safety**	
D1	Think about your unit/work area When an error is identified and corrected before the patient is affected How often is it reported?
D2	Think about your unit/work area When an error affects the patient without causing harm How often is it reported?
**Dimension 7: Support given by administrators for patient safety**	
F1	The hospital's mission demonstrates that patient safety is paramount.
F2	Hospital management provides human capital, material and financial resources necessary to improve patient safety.
F3	Hospital management is interested in patient safety after an adverse event occurs.

[1] *Item* indicates the question of the HSOPS version 2.0 questionnaire; [2] *Description* indicates the wording of the item in the final translated version and adapted to the Chilean idiosyncrasy
